# Meta-Analyses of the *5-HTTLPR* Polymorphisms and Post-Traumatic Stress Disorder

**DOI:** 10.1371/journal.pone.0066227

**Published:** 2013-06-25

**Authors:** Fernando Navarro-Mateu, Teresa Escámez, Karestan C. Koenen, Jordi Alonso, Julio Sánchez-Meca

**Affiliations:** 1 Unidad de Docencia, Investigación y Formación en Salud Mental (UDIF-SM) Subdirección General de Salud Mental y Asistencia Psiquiátrica, Servicio Murciano de Salud and CIBER de Epidemiología y Salud Pública (CIBERESP), Murcia, Spain; 2 IMIB BIOBANC-MUR, Biobanco-HUVA-AECC-FFIS, Fundación para la Formación e Investigación Sanitarias (FFIS) de la Región de Murcia, Murcia, Spain; 3 Department of Epidemiology, Mailman School of Public Health, Columbia University, New York, New York, United States of America; 4 IMIM-Institut Hospital del Mar dlnvestigacions Médiques and CIBER de Epidemiología y Salud Pública (CIBERESP), Barcelona, Spain; 5 Departamento de Psicología Básica y Metodología, Facultad de Psicología, Universidad de Murcia, Murcia, Spain; Maastricht University Medical Centre, The Netherlands

## Abstract

**Objective:**

To conduct a meta-analysis of all published genetic association studies of *5-HTTLPR* polymorphisms performed in PTSD cases

**Methods Data Sources:**

Potential studies were identified through PubMed/MEDLINE, EMBASE, Web of Science databases (Web of Knowledge, WoK), PsychINFO, PsychArticles and HuGeNet (Human Genome Epidemiology Network) up until December 2011. **Study Selection**: Published observational studies reporting genotype or allele frequencies of this genetic factor in PTSD cases and in non-PTSD controls were all considered eligible for inclusion in this systematic review. **Data Extraction**: Two reviewers selected studies for possible inclusion and extracted data independently following a standardized protocol. **Statistical analysis**: A biallelic and a triallelic meta-analysis, including the total *S* and *S*' frequencies, the dominant (*S+/LL* and *S*'*+/L*'*L*') and the recessive model (*SS/L+* and *S*'*S*'*/L*'*+*), was performed with a random-effect model to calculate the pooled OR and its corresponding 95% CI. Forest plots and Cochran's Q-Statistic and I^2^ index were calculated to check for heterogeneity. Subgroup analyses and meta-regression were carried out to analyze potential moderators. Publication bias and quality of reporting were also analyzed.

**Results:**

13 studies met our inclusion criteria, providing a total sample of 1874 patients with PTSD and 7785 controls in the biallelic meta-analyses and 627 and 3524, respectively, in the triallelic. None of the meta-analyses showed evidence of an association between *5-HTTLPR* and PTSD but several characteristics (exposure to the same principal stressor for PTSD cases and controls, adjustment for potential confounding variables, blind assessment, study design, type of PTSD, ethnic distribution and Total Quality Score) influenced the results in subgroup analyses and meta-regression. There was no evidence of potential publication bias.

**Conclusions:**

Current evidence does not support a direct effect of *5-HTTLPR* polymorphisms on PTSD. Further analyses of gene-environment interactions, epigenetic modulation and new studies with large samples and/or meta-analyses are required.

## Background

Post-traumatic stress disorder (PTSD) is a mental disorder that occurs following exposure to a potentially traumatic life event (Criterion A) and is characterized by symptoms of re-experience, avoidance, dulling of the senses and hyperarousal. Those who develop PTSD are at substantially increased risk of unemployment, marital instability and health problems (major depression, substance dependence, impaired role functioning and reduced life course opportunities) [1]. Therefore, PTSD constitutes a potential major health care burden. However, while trauma exposure rates vary between 40–80% over the course of the life of individuals, only a percentage of those exposed to traumatic events (25% approximately) will develop PTSD [2].

Recent data indicate that both the risk of trauma exposure and PTSD may be influenced by genetic factors [3], [4]. The evidence for genetic influences on risk for PTSD comes from a diversity of family, twin and molecular genetic studies. An elevated risk of PTSD among relatives with PTSD and a heritability of almost 30% suggest that genes are important risk factors in the etiology of the disorder. Molecular genetic studies using case-control candidate gene-association designs have shown a variety of results. Several candidate genes related to the current understanding of the neurobiology of the disorder have been studied [3]. The most frequently investigated genetic variant recently studied is the role of the human serotonin transporter (*5-hydroxytryptamine transporter, 5-HTT*) gene (*SLC6A4*), through polymorphisms in its promoter region. The importance of this gene is primarily due to the increasing evidence of its role in modulating sensitivity to stress and vulnerability to psychopathology [5]. The promoter activity of the *5-HTT* gene, located at *17q11.1-q12*, is modified by sequential elements within the proximal 5 regulatory region, designated the *5-HTT* gene-linked polymorphic region (*5-HTTLPR*) [6]. The less frequent short (*S*) allele in the *5-HTTLPR* is associated with lower transcriptional efficiency of the promoter compared with the more frequent long (*L*) allele [7] and has been related to suicidal behavior [8], depression [9], neurotic personality trait [7], [10], alcoholism [11] and PTSD [12].

Other *5-HTTLPR* variants have been described in a Japanese study [13] showing a third functional allele, *L_G_*, with an *A*>*G* polymorphism at position 6 of the first two 22-bp imperfect repeats that define the 16-repeat L allele (SNP rs25531). This *L_G_* is equivalent in expression to the *S* allele [11]. Thus, *5-HTTLPR* is a triallelic locus with alleles designated as *L_G_*, *L_A_*, and S and the three of them appear to act codominantly [11]. Because the *L_G_* and *S* alleles have comparable levels of serotonin transporter expression, both of which are lower than that of *L_A_*, a novel approach has been used with a reclassification of the alleles on the basis of lower and higher levels of expression [14], [15]. This approach reclassified *L_G_* and *S* as *S*' and *L_A_* as *L*'.

Differences in ethnic distribution have been described with a higher frequency of the long variant allele (*L*) in Europeans (57%; 95% CI: 49.9–61.8%) than in Asians (27%; 95% CI: 23.9–32.9%) [8] and a different distribution of the triallelic classification between Afro-American adults (L'L': 34.8%; *S*'*L*': 47.8%; and *S*'*S*': 17.4%) and European-Americans (*L*'*L*': 25.1%; *S*'*L*': 54.9%; and *S*'*S*': 20.0%) [16]. The allelic frequency of *L_G_* is 0.09–0.14 in Caucasians and 0.24 in Afro-Americans [11].

Two studies have reported an association of PTSD with the *SS* genotype [17], [18] and a third [19] reported a significant association with the *L_A_L_A_* but not with the *SS* genotype. Other studies [12], [16], [19]–[21] have not found a direct link between the *5-HTTLPR* genotype and the risk of PTSD but have reported a gene-environment (GxE) interaction whereby the genetic effect of 5-HTTLPR is modified by the level of trauma exposure [4], [22]. Of the five studies that analyzed a potential GxE interaction, four found a significant interaction with the low-expression genotype [12], [16], [17], [19], [20] and another [19] found a significant interaction but with the *L_A_L_A_* genotype. These inconsistencies in the published findings of genetic association studies of *5-HTTLPR* and PTSD might be the result of several factors that need to be systematically assessed, including different designs, statistical power and quality characteristics of primary studies.

Although previous reviews of the genetic factors involved in PTSD have been published [3], [4], [22], [23], none of them can be considered a systematic review and no meta-analysis has been conducted [24]. The main aim of the current study was to perform a systematic review and a meta-analysis of all available genetic association studies of 5-HTTLPR polymorphisms in PTSD and to determine if the *S* allele or *SS* genotype increases the risk of PTSD in those exposed to a traumatic experience compared to the *L* allele or *LL* genotype. A secondary aim of this meta-analysis was to compare the association between the genotype using the biallelic and the triallelic models and to assess if the latter better captures the effect of the *5-HTTLPR* variation on PTSD.

## Methods

### Search strategy

Potential published studies were identified through PubMed/MEDLINE, EMBASE, Web of Science databases (Web of Knowledge, WoK), PsychINFO, PsychArticles and HuGeNet (Human Genome Epidemiology Network) using the search terms: “[PTSD OR posttraumatic stress disorder OR trauma* stress] AND [ ***5-HTTLPR*** **OR** ***5-HTT*** **OR** ***SLC6A4*** **OR SERTPR OR Serotonin Transporter Gene]**” up until December 2011. The reference lists of original studies and review articles included were manually searched to identify other potentially eligible studies. In addition, the MEDLINE option `Related Articles` was used for the same purpose. To minimize potential publication bias, no restrictions were placed on time period, sample size, population and language of publication or type of report.

### Inclusion and exclusion criteria

Published observational studies (criterion 1) reporting genotype or allele frequencies of the genetic factor (criterion 2) in PTSD cases and in non-PTSD controls (criterion 3) were all considered eligible for inclusion in this systematic review. Case status was defined as having a current or one-year DSM-III, DSM-IIIR or DSM-IV (Diagnostic and Statistical Manual of Mental Disorders) diagnosis of PTSD assessed by established psychiatric interviews. Studies of all ethnic groups were considered eligible. Reviews, case-only studies, family-based designs and population studies with only healthy subjects were excluded, as well as other studies describing genetic effects on other anxiety or depression-related phenotypes such as anxiety, depression or on different personality traits.

Two review authors (MTE and FNM) independently selected studies for possible inclusion in the study. Firstly, titles and abstracts identified from the search were independently reviewed. Secondly, each review author independently examined the full text of all studies that they considered to be of possible relevance. Each review author compiled a list of studies that they believed met the inclusion criteria. The content of each review author's list was compared and any discrepancies discussed. Any disagreement was resolved by discussion and a consensus was reached by all authors.

### Data extraction

Two investigators (FNM and MTE) independently extracted data using a standardized data extraction form and they were entered into separate databases by each of the reviewers independently following standardized procedures. In case of disagreement, a consensus was reached by the reviewers. Where more than one psychiatric disorder was described in a particular study, only data from the PTSD sample were extracted. Therefore, in the case of multiple papers from a single study, only the results of the publication with the highest number of participants were included, since the unit of analysis was the study rather than the reports to avoid duplicity. When essential data were missing from the study report, the corresponding author was contacted and asked to provide the required data.

Information on the data extraction form included: author(s), journal and year of publication; methodology details (study design, sample size for both cases and controls, diagnostic tools for determination of case status and definition of case status); and sample characteristics (gender ratio, mean age, ethnic background, Hardy-Weinberg Equilibrium (HWE) in controls, genotype and allele frequencies when appropriate, type of trauma event, time from trauma exposure, severity of PTSD, other mental health disorders, including comorbid substance abuse and suicide-related behaviors).

### Quality of the studies

As poor reporting quality has been associated with a biased estimation of effects in clinical intervention studies [25], the quality of each study selected for inclusion was assessed by applying a 12-item quality checklist, derived from the STREGA statement (Strengthening the Reporting of Genetic Association Studies) [26] and other criteria [24], [27], [28]. Specifically, the quality criteria were: (1) representativeness of cases; (2) representativeness of controls; (3) same PTSD diagnostic instrument used for cases and controls; (4) trauma-exposed controls; (5) identical trauma exposure for cases and controls; (6) assessment of ethnicity; (7) blind assessment of genotyping and phenotyping; (8) quality control procedures for genotyping methods; (9) HWE testing; (10) adjustment for potential confounding variables; (11) control for multiple comparisons and (12) assessment of psychiatric comorbidity. A quality score of one was assigned if the criteria were correctly assessed and the Total Quality Score (TQS) of each study was calculated by adding all the corresponding quality item scores (range: 0–12 with a higher score indicating a higher overall quality). Discrepancies in the quality evaluation of each study were resolved by consensus. Consistent with current guidelines, we did not weight studies by TQS or exclude studies with low-quality scores.

### Statistical analysis

Inter-rater agreement was measured by Cohen's kappa coefficient for inclusion and exclusion criteria. The association between the *5-HTLLPR* allelic frequency and PTSD was examined by statistical analyses, firstly using the biallelic model (Biallelic Frequency Model, BFM) and then a triallelic one (Triallelic Frequency Model, TFM) by calculating the respective Odds Ratio (OR) and its corresponding 95% confidence interval (95% CI) as the effect size measurement for these analyses. The triallelic model was used whenever the original researchers provided the frequencies of *S*' (as the sum of the frequencies of *L_G_* and *S*) and the frequency of *L*' (*L_A_*). However, as the type of inheritance of the *5-HTTLPR* polymorphism was not yet known, two more ORs were calculated per study and model, the first one reflecting the risk of PTSD associated with possessing at least one S or S' allele (Biallelic or Triallelic Dominant Model, BDM or TDM, respectively) and the second one reflecting the risk of association with two *S* or *S*' alleles (Biallelic or Triallelic Recessive Model, BRM or TRM respectively). ORs were calculated for each study so that an OR of greater than one reflected a higher risk for PTSD. To assess the possible differences between a biallelic or a triallelic model and due to the non-independent nature of the studies compared, a qualitative comparison of the results was performed on those studies which had data for both models in the same sample.

Random-effects models were applied in the statistical analyses because heterogeneity among the studies was expected. This assumes a genuine diversity in the results of the various studies and it incorporates a between-studies variance into the calculations. In each meta-analysis, a pooled OR and its corresponding 95% CI were calculated. In addition, the statistical significance of the pooled OR was assessed using the Z test [29]. A sensitivity analysis was performed to assess whether our results were substantially influenced by the presence of any individual study by systematically removing each study and recalculating the significance of the result.

Forest plots were constructed to represent the individual and pooled effect estimates, with their 95% CIs, and to allow visual inspection for study heterogeneity. To check for this among the studies, both the Cochran's Q-statistic and I^2^ index were calculated [30]. When the ORs are homogeneous, Q-statistic follows a chi-squared distribution with k – 1 degrees of freedom (d.f.) (k being the number of studies). The degree of heterogeneity was estimated with the I^2^ index (I^2^ = 100×(Q–d.f.)/Q), which can be interpreted as the percentage of total variation across the studies due to their different characteristics. I^2^ values around 25%, 50%, and 75% denote low, moderate and large heterogeneity, respectively.

To explore heterogeneity, different subgroup analyses, which allow chi-squared tests for between group differences, were performed taking as potential moderators the quality items, ethnicity and study design. In addition, meta-regression analyses were carried out with continuous moderators, such as the mean age of participants, the TQS, percentage of males, year of publication, percentage of Caucasian or Euro-Americans and percentage of Afro-Americans or Africans to test potential explanatory variables leading to heterogeneity.

Chi-squared tests were conducted to test for HWE in the reported genotype frequencies among the controls in case-control and cross-sectional studies and among the whole population in the cohort studies. Deviations from HWE (P-value <0.05) might indicate genotyping errors, limited population size, population substructure or newly occurring mutations. No deviation from HWE is considered a quality measure of a genetic association study and a subgroup analysis was performed to compare those studies with and without HWE.

To assess whether publication bias may be a threat to the validity of the pooled ORs, funnel plots with the Duval and Tweedie's trim-and-fill method [31] were applied, as well as the Egger test [32]. Where funnel plot asymmetry was observed, effect estimates corrected for small study effects, such as publication bias, were generated by the trim-and-fill method. This uses available data to estimate the number and outcome of missing (unreported) studies and recalculates the overall effect that would be observed with their inclusion. The Egger test is an unweighted regression consisting of taking the precision of each study as the independent variable (precision being defined as the inverse of the standard error of each effect size) and the effect size divided by its standard error as the dependent variable. A non-statistically significant result of the t-test for the hypothesis of an intercept equal to zero allows discounting of publication bias as a threat to the validity of the pooled effect [32].

All statistical tests were interpreted assuming a significance level of 5% (α = 0.05) and all were two-tailed. The main statistical analyses were carried out using the software package RevMan 5.029. Meta-regressions, funnel plots with the trim-and-fill method and the Egger test were calculated with the program *Comprehensive Meta-analysis* 2.0 [33]. Methods of the analysis and inclusion-exclusion criteria were specified a priori and documented in a protocol. The published recommendations for systematic reviews of genetic association studies were followed [24], [27], [34]. Since we only used previously published data, we did not consider it necessary to seek ethical approval or written informed consent.

## Results

**Figure 1:**
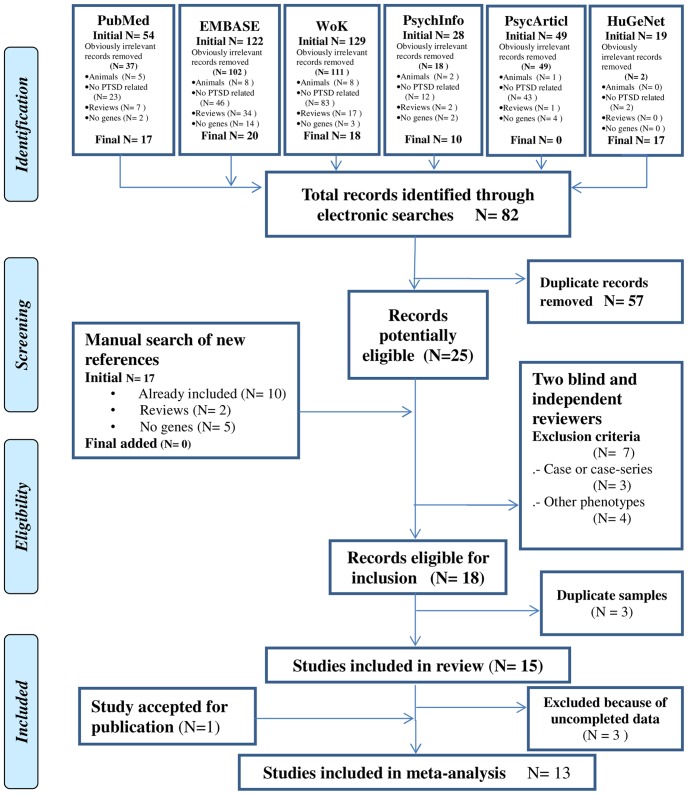
Flow chart of the Meta-Analysis of *5-HTTLPR* polymorphisms and Post-traumatic Stress Disorder (PTSD). Adapted from Sagoo GS, Little J, Higgins JP, Systematic Reviews of Genetic Association Studies. Human Genome Epidemiology Network. PLOS Med 2009; 6(3): e28 doi: e28 10.1371/journal.pmed.1000028 and Moher D, Liberati A, Tetzlaff J, Altman DG, The PRISMA Group (2009). Preferred Reporting Items for Systematic Reviews and Meta-Analyses: The PRISMA Statement. PLoS Med 6(6): e1000097. doi:10.1371/journal.pmed1000097.

Figure 1 details the search process flow and results. From a total of 25 potentially eligible studies, seven were excluded because they were a case or case-series design [35]–[37] or were focused on other phenotypes apart from PTSD [38]–[41]. Cohen's kappa inter-rater agreement coefficient for the three inclusion criteria ranged from 0.70 (criterion 3) to 1.0 (criteria 1 and 2) and 0.905 for the exclusion criteria. From the 18 studies eligible for inclusion, three were excluded as they were based on the same sample ([12], [42] and [43]) as the final included studies ([16] and [44]), respectively. Fifteen studies were included in the review and their data were extracted [16]–[21], [45]–[50]. An additional study was sent by the corresponding author who was contacted for further information about a published study. By the time this meta-analysis was performed, this study was accepted for publication and, therefore, included in this review [51]. Three studies were excluded as essential data on *5-HTTLPR* genotypic frequencies could not be provided by the authors [44], [52], [53]. Finally, 13 studies were included in the meta-analysis.

The characteristics of studies eligible for inclusion are described in Table 1, including year of publication, study design, number of PTSD cases, controls and total sample, number and percentage of males, mean age, diagnostic instrument used to assess PTSD, type of PTSD assessed, biallelic or triallelic genotype approach, whether a GxE interaction had been analyzed and a brief description of the environment studied and of the final decision to include or exclude. The pooled population was 1,874 patients with PTSD and 7,785 controls in the biallelic meta-analysis and 627 and 3,524, respectively, in the triallelic one.

**Table 1:** Characteristics of association studies eligible for inclusion in Meta-Analysis of *5-HTTLPR* polymorphisms and Post-traumatic Stress Disorder (PTSD).

First author	Year	Study design		PTSD N	Controls N	Total sample (N)	Males N (%)	MeanAge	Diagnostic instrument	PTSD assessed	Biallelic/ Triallelic Genotype	GxE study	Interaction description	Remarks
Lee [18]	2005	Case-Control		100	197	297	119 (40.1)	34.94	SCID-I Korean Version	Current	Biallelic	No	-	Included
Koenen [16]	2009	Cross-Sectional		19	571	590	215 (36.4)	65.1	National Women's Study PTSD Module	Last 6 months	Triallelic	Yes	High crime & High unemployment rate	Included
Mellman [21]	2009	Case-Control		55	63	118	40 (33.9)	39.97	CAPS	Lifetime	Triallelic	No	-	Included
Xie [20]	2009	Cross-Sectional		229	1023	1252	656 (52.4)	38.97	SSADDA	Lifetime	Triallelic	Yes	Childhood adversity & Adult traumatic events	Included
Grabe [19]	2009	Cross-Sectional		67	1596	1663	857 (51.5)	57.61	PTSD Module of SCID	Lifetime	Triallelic	Yes	Frequent Trauma event	Included
Thakur [47]	2009	Cohort study		24	17	41	19 (46.3)	31.96	CAPS	Last year	Biallelic	No	–	Included
King [52]	2009	Cohort study		–	–	378	–	–	Computer-aided telephone interviews.	Lifetime	Triallelic	Yes	Childhood adversity	Not included
Kolassa [17]	2010	Cross-Sectional		331	77	408	218 (53.4)	34.72	Post-traumatic Diagnostic Scale	Lifetime	Biallelic	Yes	Number of traumatic events experienced	Included
Handwerger [53]	2010	Case-Control		18	18	36	–	–	–	–	–	No	–	Not included
Saying [48]	2010	Cohort study		29	48	77	47 (61)	–	CAPS	Lifetime	Biallelic	No	–	Included
Koenen [50]	2011	Cross-Sectional		23	77	100	40 (40)	45.32	Modified PTSD Checklist	Lifetime	Triallelic	Yes	Number of traumatic events	Included
Morey [49]	2011	Case-Control		22	20	42	22 (52.4)	34.04	DTS. Cutoff score = 32	Current	Triallelic	No	–	Included
Valente [46]	2011	Case-Control		65	34	99	28 (28.3)	39.99	SCID-I & CAPS	Current	Biallelic	No	–	Included
Wang [45]	2011	Case-Control		212	176	388	200 (51.55)	49.06	SCID-I, MINI, CES y CAPS	Current	Triallelic	No	–	Included
King [44]	2011	Case-Control		16	14	30	–	–	–	–	Biallelic	No	–	Not included
Xie [51]	2012	Cross- Sectional	AA	321	2078	2399	1343 (56)	42.2	SSADDA	Lifetime	Biallelic	Yes	Childhood adversity	Included
			EA	398	2381	2779	1559 (56.1)	39						

GxE study: Gene-Environmental interaction study; SCID-1: Structured Clincal Interview for DSM-IV; CAPS: Clinical Assessed PTSD Scale; SSADDA: Semi-Structured Assessment for Drug Dependence and Alcoholism; MINI: Mini-International Neuropsychiatric Interview; CES: Combat Exposure Scale; DTS: Davidson Trauma Scale; AA: Afro-Americans; EA: Euro-Americans.

Originally, six studies [17], [18], [46]–[48], [51] described a biallelic analysis and seven [16], [19]–[21], [45], [49], [50] a triallelic. Nevertheless, five of the latter group [19], [21], [45], [49], [50] provided the frequencies of the biallelic genotype so that they could also be included in the meta-analysis of the biallelic approach (Table 2).

**Table 2:** Frequencies of the *5-HTTLPR* alleles and polymorphisms of the included studies&.

		Biallelic genotype approach										HWE	Triallelic genotype approach										HWE
		PTSD					Controls					P-value	PTSD					Controls					P-value
		S	L	SS	SL	LL	S	L	SS	SL	LL		S'	L'	S'S'	S'L'	L'L'	S’	L'	SvS'	S'Lv	LvL'	
Lee [18]	2005	175	25	77	21	2	319	75	129	61	7	0.81	–	–	–	–	–	–	–	–	–	–	–
Koenen [16]	2009	–	–	–	–	–	–	–	–	–	–	–	17	21	4	9	6	539	603	116	307	148	0.06
Mellman [21	2009	42	70	5	32	19	35	93	7	21	36	0.16	66	46	20	26	10	67	61	18	31	15	0.81
Xie [20]	2009	–	–	–	–	–	–	–	–	–	–	–	234	224	58	118	53	957	1089	234	489	300	0.20
Grabe [19]	2009	40	94	6	28	33	1294	1898	264	766	566	0.86	48	86	8	32	27	1512	1680	364	784	448	0.55
Thakur [47]	2009	23	25	8	7	9	22	12	7	8	2	0.09	–	–	–	–	–	–	–	–	–	–	–
Kolassa [17]	2010	112	550	15	82	234	29	125	1	27	49	0.20	–	–	–	–	–	–	–	–	–	–	–
Saying [48]	2010	30	28	6	18	5	41	55	6	29	13	0.045	–	–	–	–	–	–	–	–	–	–	–
Koenen [50]	2011	10	34	1	8	13	45	103	12	21	41	0.005	21	23	4	13	5	67	81	17	33	24	0.39
Morey [49]	2011	22	22	7	8	7	18	22	3	12	5	0.34	26	18	10	6	6	22	18	5	12	3	0.34
Valente [46]	2011	55	71	13	29	21	38	30	9	20	5	0.26	–	–	–	–	–	–	–	–	–	–	–
Wang [45]	2011	207	217	56	95	61	131	221	21	89	66	0.28	261	163	94	73	45	164	188	29	106	41	0.005
Xie (AA) [51]	2012	222	650	26	170	240	1269	3997	170	929	1534	0.07	–	–	–	–	–	–	–	–	–	–	–
Xie (EA) [51]	2012	460	564	96	268	148	2511	3187	566	1379	904	0.34	–	–	–	–	–	–	–	–	–	–	–

*S*, short allele; *L*, long allele; *S*' as *S* + *L_G_*; and *L*' as *L_A._*

& Dominant model (*S+* vs *LL* or *S*'+vs L'L'): *S+* (or *S*'*+*) genotype frequencies are calculated as the sum of *SS* (or *S*'*S*') and *SL* (or *S*'*L*') frequencies. Recessive model (*SS* vs *L+* or *S*'*S*' vs *L*'*+*): *L+* (or *L*'+) genotype frequencies are calculated by the sum of *LL* (or *L*'*L*') and *SL* (or *SvL*') genotype frequencies.

### Meta-analysis of the allelic association with PTSD

**Figure 2:**
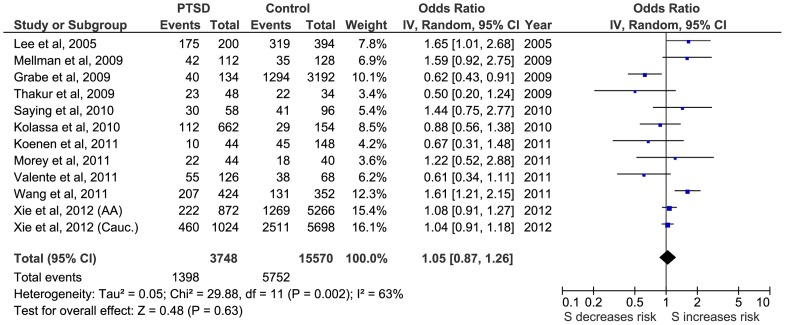
Forest plot of the 5-HTTLPR biallelic frequency model (S vs L) and Post-traumatic Stress Disorder (PTSD).

**Figure 3:**
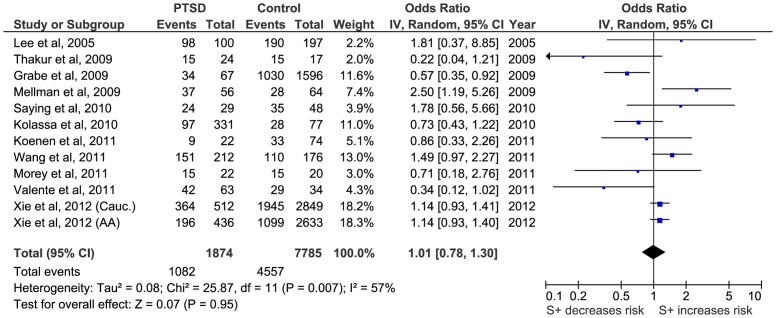
Forest plot of *5-HTTLPR* biallelic dominant model (*S*+ vs *LL*) and Post-traumatic Stress Disorder (PTSD).

**Figure 4:**
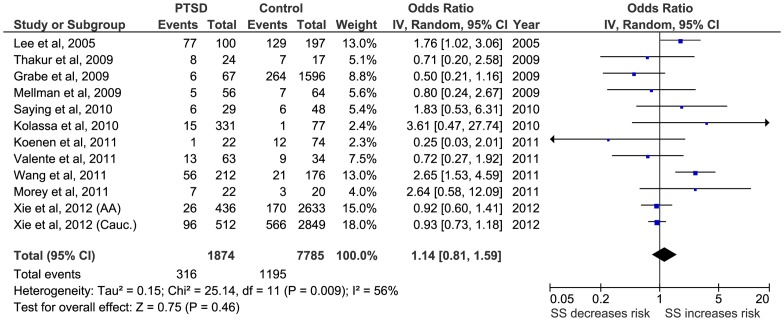
Forest plot of *5-HTTLPR* biallelic recessive model (*SS* vs *L*+) and Post-traumatic Stress Disorder (PTSD).

Firstly, a meta-analysis of the biallelic approach was performed for: i) allele frequency (*S vs L*) (Fig 2); ii) the dominant model, BDM (*S+vs LL*) (Fig 3); and iii) the recessive model, BRM (*SS vs L+*) (fig 4). The overall association between genotypes and the risk of PTSD was not significant using all three different approaches: (i) OR  = 1.05; 95% CI  = 0.87, 1.26; p = 0.63; I^2^ = 63; ii) OR  = 1.01; 95% CI  = 0.78, 1.30; p = 0.95; I^2^ = 57; and iii) OR  = 1.15; 95% CI  = 0.82, 1.60; p = 0.41; I^2^  = 55, respectively) (Figs 2, 3 and 4).

**Figure 5:**
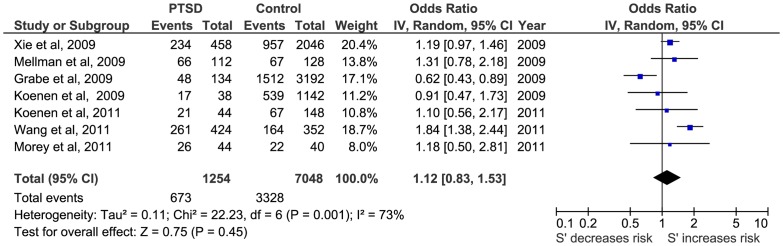
Forest plot of 5-HTTLPR triallelic frequency model (*S*' vs *L*') and Post-traumatic Stress Disorder (PTSD).

**Figure 6:**
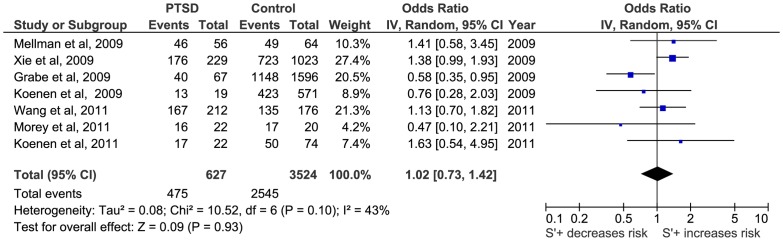
Forest plot of *5-HTTLPR* triallelic dominant model (*S*'+ vs *L*'*L*') and Post-traumatic Stress Disorder (PTSD).

**Figure 7:**
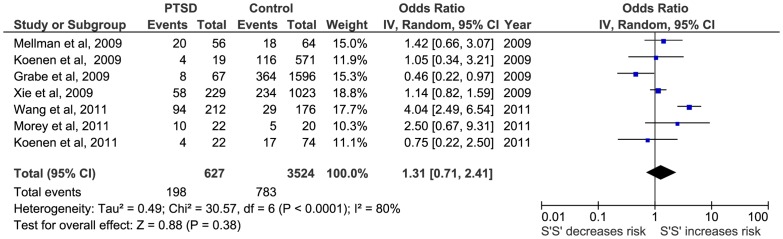
Forest plot of *5-HTTLPR* triallelic recessive model (*S*'*S*' vs *L*'+) and Post-traumatic Stress Disorder (PTSD).

Secondly, a meta-analysis of the triallelic approach offered similar results for the TFM model (OR  = 1.15; 95%CI  = 0.82, 1.60; p = 0.41; I^2^ = 74), for TDM model (OR  = 1.02; 95%CI  = 0.73, 1.48; p = 0.93; I^2^ = 43) and for the TRM (OR  = 1.31; 95% CI  = 71, 2.41; p = 0.38; I^2^ = 80) (Figs 5, 6 and 7, respectively).

### Comparison of the biallelic or triallelic models of the 5-HTTLPR polymorphisms

Of the seven studies describing a triallelic approach, it was not possible to calculate the biallelic frequencies of one of the studies and we could not obtain further data from the authors on request [16]. The biallelic frequencies of the second study [20] were included in a different study when requested [51] and the genotype frequencies from PTSD and controls could not be calculated from the text as they only provided the total biallelic frequencies. Finally, five studies [19], [21], [45], [49], [50] reported allele and *5-HTTLPR* polymorphism frequencies of the same population allowing comparison of both approaches. The result were very similar in the allele frequency model (BFM, OR  = 1.07; 95% CI: 0.66, 1.74; I^2^ = 78% and TFM, OR  = 1.14; 95% CI: 0.70, 1.87; I^2^ = 81%), in the dominant model (BDM, OR =  1.09; 95% CI: 0.61, 1.96; I^2^  =  72%, TDM, OR = 0.93; 95% CI: 0.60, 1.44; I^2^  =  39%) and in the recessive model (BRM, OR  = 1.05; 95% CI: 0.41, 2.67; I^2^ = 73%, TRM, OR  = 1.40; 95% CI: 0.54, 3.57; I^2^ = 85%).

### Sensitivity analysis

Only one study [19] was found to alter the results when individually removed from the meta-analysis during sensitivity analysis and it affected the TFM model (OR  = 1.31; 95% CI  = 1.06, 1.63; p = 0.01; I^2^ = 35). The remaining results were robust to sensitivity analysis, with the overall P-values remaining non-significant when each study was individually removed from the analysis. Analysis of heterogeneity varied from moderate (I^2^ = 43 in TDM) to extreme (I^2^ = 80 in TRM) in the different models.

### Publication bias

**Figure 8:**
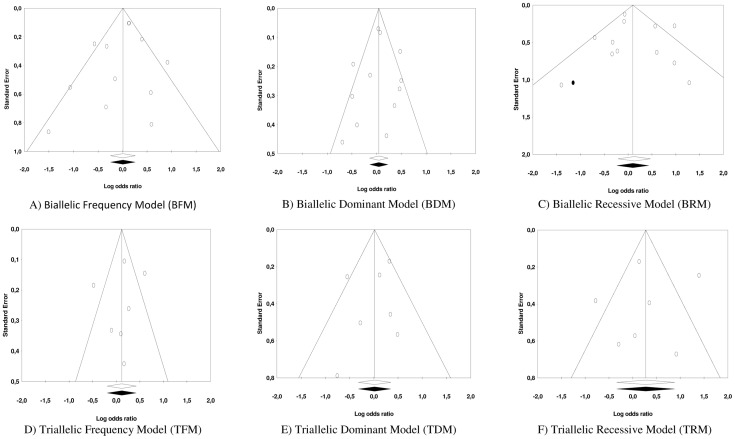
Funnel plots of *5-HTTLPR* polymorphisms and Post-traumatic Stress Disorder (PTSD) to assess publication bias. **White circles** represent each of the included studies. **Black circles** represent the new effect estimated to achieve symmetry.

In order to assess whether publication bias might be a threat to the pooled ORs, several graphical and analytic techniques were applied. Firstly, funnel plots were constructed and the trim-and-fill method proposed by Duval and Tweedie was applied to them in order to achieve symmetry when they showed an asymmetric pattern. Of the six funnel plots constructed, the trim-and-fill method did not require the addition of any new effect estimate in five of them to achieve symmetry (Fig 8). The only exception was with the Biallelic Recessive model where the Duval and Tweedie's method added a new effect estimate. However, the adjusted pooled OR (OR  = 1.10; 95% CI: 0.79, 1.54) showed a negligible difference from the original pooled OR (OR  = 1.14; 95% CI: 0.81, 1.59). In addition, the Egger test was applied to each of the six meta-analytic databases, in all cases achieving a non-statistically significant result for the intercept of the regression model (*p*>0.05) (Table 3). Therefore, on the basis of this, we felt that we could reasonably discount publication bias as a threat to our meta-analytic findings.

**Table 3:** Analyses of publication bias by the Egger test.

Model	Intercept	SE	T	df	p-value
Biallelic					
BFM	−0.319	0.857	−0.371	10	0.718
BDM	−0.658	0.721	−0.912	10	0.383
BRM	0.273	0.763	0.358	10	0.727
Triallelic					
TFM	−0.842	1.738	−0.484	5	0.648
TDM	−0.775	1.160	−0.668	5	0.534
TRM	−0.625	2.010	−0.311	5	0.768

SE: Standard error; T: T-test; df: Degrees of freedom. BFM: Biallelic Frequency Model; BDM: Biallelic Dominant Model; BRM: Biallelic Recessive Model; TFM: Triallelic Frequency Model; TDM: Triallelic Dominant Model; TRM: Triallelic Recessive Model.

### Quality of the studies

Table 4 describes the quality characteristics of the studies analyzed. Included studies (N = 13) had a superior TQS (mean  = 6.31, SD  = 2.428) than those excluded studies (N = 3; mean  = 1.33; SD  = 1.528) (p = 0.005). The TQS of those studies with a biallelic approach (N = 6; mean  = 4.17, SD  = 1.329) was significantly lower than of those with a triallelic approach (N = 7; mean  = 8.14; SD  = 1.345) (p<0.001).

**Table 4:** Description of the quality characteristics of the included and excluded studies in the Meta-Analysis of *5-HTTLPR* polymorphisms and Post-traumatic Stress Disorder (PTSD).

First author	Year	1		2			3		4			5			6		7	8	9	10	11	12	
		Repre sentative cases$		Repre sentative controls#			Same diagnostic instrument		Trauma Exposed controls			Identical trauma experience			Ethnicity&		Blind assessment	Quality control Genotyping	HWE† testing	Confounders adjustment	Control for multiple comparisons	Psychiatric Comorbidity assessed	TQS ^‡^
INCLUDED STUDIES																							
Lee [18]	2005		No		No			No		No		No			AS (100)		No	No	Yes	No	No	No	2
Koenen [16]	2009		No		Yes			Yes		Yes		Yes			EA (90.7) Others (9.5)		No	Yes	No	Yes	No	No	7
Mellman [21]	2009		No		Yes			Yes		Yes		No			AA (100)		No	Yes	No	Yes	Yes	Yes	8
Xie [20]	2009		No		Yes			Yes		Yes		No			AA (53.5) EA (46.5)		No	No	Yes	Yes	No	Yes	7
Grabe [19]	2009		No		Yes			Yes		Yes		No			Eu (100)		No	Ye	Yes	Yes	No	Yes	8
Thakur [47]	2009		No		Yes			Yes		Yes		No			Caucasian (95.1) Other (4.9)		Yes	No	Yes	No	No	No	5
Kolassa [17]	2010		No		Yes			Yes		Yes		No			Af (100)		No	No	Yes	No	No	No	5
Saying [48]	2010		No		Yes			Yes		Yes		No			No		No	No	No	Yes	No	Yes	5
Koenen [50]	2011		Yes		Yes			Yes		Yes		No			AA (79) Others (21)		No	Yes	No	Yes	No	Yes	8
Morey [49]	2011		No		Yes			Yes		Yes		Yes			Caucasian (47.6) Others (52.4)		No	No	Yes	No	Yes	Yes	8
Valente [46]	2011		No		No			Yes		Mix		No			No		No	No	Yes	No	No	Yes	3
Wang [45]	2011		No		Yes			Yes		Yes		Yes			Caucasian (71.5) AA (24) Others (4.5)		Yes	Yes	Yes	Yes	Yes	Yes	11
Xie [51]	2012		No		No			Yes		No		No			AA (46.3) EA (53.7)		No	No	Yes	Yes	No	Yes	5
EXCLUDED STUDIES																							
King [52]	2009	No				No					Yes		Mix	No		AA, EA	No	No	No	Yes	No	No	3
Handwerger [53]	2010	No				No					–		Yes	–		–	No	No	No	No	No	No	1
King [44]	2011	No				No					–		Mix	No		–	No	No	No	No	No	No	0

$ Representativeness of cases included all eligible cases with outcome of interest over a defined period of time, all cases in a defined catchment area, all cases in a defined hospital or clinic, group of hospitals, health maintenance organization, or an appropriate sample of those cases (e.g. random sample).

# Representativeness of controls assesses whether the control series used in the study is derived from the same population as the cases and essentially would have been cases had the outcome been present and included community and clinical controls within the same community or hospitalized population as cases.

& If Yes, the % of the different ethnics groups are provided in brackets.AS: Asiatic. EA: Euro-American; AA: Afro-Americans; Af: African; Eu: Europeans.

† HWE: Hardy-Weinberg Equilibrium; ‡TQS: Total Quality Score.

The subgroup analysis of the different quality components (Tables 5 and 6) showed that there were some significant differences in estimated effect size between those studies that fulfilled the quality criteria and those that did not: the same principal trauma for PTSD and controls (BFM and BRM), reporting of HWE (BDM) and control for multiple comparisons (BFM and BDM) in the biallelic analysis. In the triallelic analysis, the significant differences were for the same principal trauma for PTSD and controls (TFM and TRM), the presence of blind assessment (TRM) and control for multiple comparisons (TFM and TRM). Moreover, when stratifying for the quality criteria, the pooled effect size of those that fulfilled them reached significance (same principal trauma for PTSD and controls – BFM, BRM and TRM – presence of blind assessment – TRM – and control for multiple comparisons – BFM, TFM and TRM -). On the other hand, there was one characteristic where not fulfilling the quality criteria significantly decreased the risk, i.e. those studies which did not use the same diagnostic instrument for PTSD and controls (BFM and BRM) (Tables 5 and 6).

**Table 5:** Subgroup analysis of study design, type of PTSD assessed and quality characteristics in the Meta-Analysis of *5-HTTLPR* polymorphisms and Post-traumatic Stress Disorder (PTSD) in the biallelic approach.

	K‡	Number of participants		Frequency Model^ #^				Dominant Model				Recessive Model			
				OR	95%CI	I^2^(%)	P-value ^&^	OR	95%CI	I^2^ (%)	P-value ^&^	OR	95%CI	I^2^ (%)	P-value ^&^
**Case representativeness**															
Yes	1		96	0.67	0.31, 1.48	NA		0.86	0.33, 2.26	NA		0.25	0.03, 2.01	NA	
No	11		9563	1.07	0.88, 1.26	63	0.27	1.02	0.78, 1.32	61	0.75	1.18	0.81, 1.65	56	0.15
Control representativeness															
Yes	8		2835	1.01	0.72, 1.43	70		0.98	0.86, 1.38	65		1.20	0.62, 2.30	50	
No	4		6824	1.06	0.88, 1.28	54	0.82	1.09	0.86, 1.38	39	0.66	1.03	0.77, 1.39	40	0.68
**Same diagnostic instrument**															
Yes	11		9362	1.01	0.84, 1.22	63		0.99	0.77, 1.29	61		1.07	0.74, 1.53	54	
No	1		297	**1.65**	**1.01, 2.68**	NA	0.07	1.81	0.37, 8.85	NA	0.47	**1.76**	**1.02, 3.06**	NA	0.13
**Trauma exposed control**															
Yes	9		2932	0.96	0.69, 1.33	70		0.89	0.57, 1.39	66		1.12	0.62, 2.02	57	
No	3		6727	1.09	0.94, 1.26	38	0.47	1.15	0.99, 1.32	0	0.28	1.08	0.76, 1.51	56	0.90
**Same principal trauma for PTSD and controls**															
Yes	2		430	**1.57**	**1.19, 2.06**	0		1.38	0.90, 2.12	2		**2.65**	**1.58, 4.44**	0	
No	10		9229	0.98	0.81, 1.26	63	**0.005**	0.96	0.72, 1.27	61	0.17	0.97	0.74, 1.26	20	**0.0007**
Assessment of ethnicity															
Yes	10		9485	1.07	0.88, 1.29	65		1.04	0.81, 1.33	57		1.15	0.79, 1.67	62	
No	2		174	0.93	0.40, 2.14	72	0.75	0.77	0.15, 3.87	76	0.72	1.06	0.43, 2.59	25	0.87
**Presence of blind assessment**															
Yes	2		429	0.98	0.31, 3.02	83		0.69	0.11, 4.29	78		1.57	0.45, 5.53	70	
No	10		9230	1.01	0.85, 1.20	52	0.95	0.99	0.76, 1.28	56	0.70	1.02	0.76, 1.36	29	0.51
**Reporting of quality control procedures in genotyping methods**															
Yes	4		2267	1.05	0.60, 1.83	84		1.15	0.60, 2.22	78		0.89	0.31, 2.56	77	
No	8		7392	1.04	0.89, 1.22	35	0.99	0.98	0.76, 1.27	41	0.66	1.09	0.83, 1.43	24	0.71
**Reporting HWE^†^**															
Yes	9		9366	1.01	0.82, 1.24	69		0.92	0.71, 1.20	60		1.18	0.81, 1.71	64	
No	3		293	1.23	0.76, 2.00	39	0.46	1.64	0.85, 3.17	33	0.11	0.90	0.34, 2.37	27	0.61
**Adjustment for potential confounders**															
Yes	7		8774	1.09	0.88, 1.35	70		1.16	0.38, 1.03	59		1.04	0.67, 1.60	67	
No	5		885	0.92	0.60, 1.41	57	0.48	0.63	0.38, 1.03	14	**0.03**	1.39	0.82, 2.35	20	0.40
**study design. In addition, meta regres**															
Yes	3		550	1.57	1.23, 2.01	0		1.60	0.97, 2.63	31		1.95	0.93, 4.07	38	
No	9		9109	0.94	0.87, 1.14	56	**0.001**	0.89	0.67, 1.16	55	**0.04**	0.98	0.73, 1.31	33	0.09
**Psychiatric comorbidity assessed**															
Yes	9		8913	1.05	0.86, 1.29	66		1.08	0.83, 1.40	59		1.07	0.73, 1.56	58	
No	3		746	0.97	0.53, 1.78	69	0.81	0.70	0.30, 1.62	36	0.34	1.57	0.87, 2.83	11	0.26
**Study design**															
Cross-Sectional	5		8597	0.94	0.78, 1.12	52		0.94	0.73, 1.21	58		0.87	0.64, 1.17	23	
Case-Control	5		944	1.32	0.93, 1.86	56		1.20	0.63, 2.28	60		1.61	0.97, 2.66	45	
Cohort	2		118	0.89	0.32, 2.49	71	0.22	0.69	0.09, 5.26	75	0.75	1.16	0.46, 2.91	6	0.12
**Type of PTSD assessed**															
Lifetime PTSD	7		8794	0.99	0.83, 1.19	50		1.04	0.74, 1.36	61		0.90	0.71, 1.14	8	
Current PTSD	4		824	1.24	0.80, 1.93	66		0.93	0.43, 2.02	56		**1.79**	**1.06, 3.01**	45	
Last year	1		41	0.50	0.20, 1.24	NA	0.21	0.22	0.04, 1.21	NA	0.21	0.71	0.20, 2.58	NA	0.06

‡ K: Number of studies; ^#^Number of alleles S or L duplicates the number of participants as each participant has two alleles (S, L or one of each one); OR: Odds Ratio; 95% CI: 95% Confident Interval; & Chi-squared test for subgroup differences; NA: Not applicable; **^†^**HWE: Hardy-Weinberg Equilibrium.

**Table 6:** Subgroup analyses of study design, type of PTSD assessed and quality characteristics in the Meta-Analysis of *5-HTTLPR* polymorphisms and Post-traumatic Stress Disorder (PTSD) in the triallelic approach.

	K‡	Number of participants			Frequency Model^#^								Dominant Model								Recessive Model					
					OR		95%CI		I^2^ (%)		P-value ^&^		OR		95%CI		I^2^ (%)		P-value ^&^		OR		95%CI		I^2^ (%)	P-val ue ^&^
Case representativeness																										
Yes	1	96			1.10		0.56, 2.17		NA				1.63		0.54, 4.95		NA				0.75		0.22, 2.50		NA	
No	6	4055			1.13		0.80, 1.58		77		0.96		0.97		0.68, 1.40		50		0.39		1.41		0.73, 2.73		83	0.37
**Control representativeness**																										
Yes	7	4151			1.09		0.80, 1.49		73				1.02		0.73, 1.42		43				1.31		0.71, 2.41		80	
No	0	0			NA		NA		NA		NA		NA		NA		NA		NA		NA		NA		NA	NA
**Same diagnostic instrument**																										
Yes	7	4151			1.12		0.83, 1.53		73				1.02		0.73, 1.42		43				1.31		0.71, 2.41		80	
No	0	0			NA		NA		NA		NA		NA		NA		NA		NA		NA		NA		NA	NA
**Trauma exposed control**																										
Yes	7	4151			1.12		0.83, 1.53		73				1.02		0.73, 1.42		43				1.31		0.71, 2.41		80	
No	0	0			NA		NA		NA		NA		NA		NA		NA		NA		NA		NA		NA	NA
**Same principal trauma for PTSD and controls**																										
Yes	3	1020		1.76			0.83, 2.31		0				0.99		0.65, 1.49		0				**2.45**		**1.05, 5.72**		59	
No	4	3131		0.99			0.72, 1.35		62		**0.006**		1.09		0.64, 1.86		66		0.75		0.92		0.57, 1.49		48	**0.05**
**Assessment of ethnicity**																										
Yes	7	4151		1.12			0.83, 1.53		73				1.02		0.73, 1.42		43				1.31		0.71, 2.41		80	
No	0	0		NA			NA		NA		NA		NA		NA		NA		NA		NA		NA		NA	NA
**Presence of blind assessment**																										
Yes	1	388		1.09			0.67, 1.77		82				1.13		0.70, 1.82		NA				**4.04**		**2.49, 6.54**		NA	
No	6	3763		1.19			0.98, 1.45		0		0.75		0.98		0.63, 1.52		52		0.67		1.02		0.69, 1.51		34	<0.0001
**Reporting of quality control procedures in genotyping methods**																										
Yes	5	2857		1.09		0.67, 1.77			82				0.94		0.63, 1.39		34				1.20		0.48, 3.05		85	
No	2	1294		1.19		0.98, 1.45			0		0.75		1.06		0.43, 2.62		44		0.81		1.29		0.74, 2.25		22	0.90
**Reporting HWE^†^**																										
Yes	4	3345		1.13		0.71, 1.79		86				0.93		0.58, 1.50		67				1.49		0.59, 3.76		90		
No	3	806		1.13		0.80, 1.59		0		0.99		1.19		0.67, 2.10		0		0.52		1.14		0.65, 2.01		0		0.63
**Adjustment for potential confounders**																										
Yes	6	4109		1.12		0.80, 1.56		78				1.05		0.74, 1.48		47				1.22		0.63, 2.35		83		
No	1	42		1.18		0.50, 2.81		NA		0.91		0.47		0.10, 2.21		NA		0.32		2.50		0.67, 9.31		NA		0.34
**Control for multiple comparisons**																										
Yes	3	550		**1.65**		**1.29, 2.09**		0				1.11		0.74, 1.67		0				**2.55**		**1.22, 5.32**		61		
No	4	3601		0.92		0.63, 1.35		69		**0.01**		0.97		0.56, 1.69		67		0.70		0.85		0.53, 1.38		40		0.01
**Psychiatric comorbidity assessed**																										
Yes	6	3561		1.15		0.82, 1.62		77				1.04		0.72, 1.51		50				1.35		0.69, 2.66		83		
No	1	590		0.91		0.47, 1.73		NA		0.52		0.76		0.28, 2.03		NA		0.55		1.05		0.34, 3.21		NA		0.70
**Study design**																										
Case-Control	4		3601	0.92		0.63, 1.35		69				0.97		0.56, 1.69		67				0.85		0.53, 1.38		40		
Cohort	3		550	1.65		1.29, 2.09		0		**0.01**		1.11		0.74, 1.67		0		0.70		**2.55**		**1.22, 5.32**		61		0.01
**Type of PTSD assessed**																										
Lifetime PTSD	4		3131	1.00		0.69, 1.45		71				1.09		0.64, 1.86		66				0.92		0.57, 1.49		48		
Current PTSD	2		430	**1.76**		**1.34, 2.31**		0				1.00		0.56, 1.79		11				**3.82**		**2.43, 6.00**		0		
Last 6 months	1		590	0.91		0.47, 1.73		NA		**0.02**		0.76		0.28, 2.03		NA		0.82		1.05		0.34, 3.21		80		<0.0001

‡ K: Number of studies; ^#^Number of alleles S or L duplicates the number of participants as each participant has two alleles (S, L or one of each one); OR: Odds Ratio; 95% CI: 95% Confident Interval; ^&^Chi-squared test for subgroup differences; NA: Not applicable; **^†^**HWE: Hardy-Weinberg Equilibrium.

The subgroup analysis of study design and the type of PTSD assessed revealed significant differences between study design in TFM and TRM and between types of PTSD assessed (TFM and TRM). The pooled risk effect of cohort studies and restriction of the analysis to current PTSD significantly increased the risk in those carrying *S*' (TFM) or *S*'*S*' (TRM) (Table 6).

Four studies did not calculate HWE (Table 4) but, when we calculated it (Table 2), two of them were in HWE [16], [21] and the rest were not [48], [50]. Another study was only in HWE in the biallelic approach but not in the triallelic one [45]. Given these discrepancies, we performed a subgroup analysis with our calculated HWE and there were significant differences in the triallelic analysis between those studies in HWE when compared to those without in the TFM (OR  = 1.00; 95% CI  = 0.76, 1.32; I^2^ = 53 and OR  = 1.84; 95% CI  = 1.38, 2.44; I^2^ = Not applicable, respectively, p-value of the Chi-squared test for subgroup difference  = 0.003) and in TRM (OR  = 1.02; 95% CI  =  0.69, 1.51; I^2^ = 34 and OR  = 4.04; 95% CI  = 2.49, 6.54; I^2^ =  Not applicable, respectively, p-value of the Chi-squared test for subgroup difference <0.0001).

Meta-regression analysis (Table 7) showed modifying effects of four variables, two of them in the direction of decreasing the risk of the *S*'*+* on PTSD (the median age of participants –TDM- and the percentage of European or Caucasian -TDM-) and the other two increasing the effect of *S*'*+* on PTSD (the percentage of Afro-Americans or Africans – TDM -) and that of *S*'*S*' (TQS – TRM -). No significant association with other pre-specified moderator variables was identified.

**Table 7:** Meta-regression analysis of potential modifying variables in the Meta-Analyses of *5-HTTLPR* polymorphisms and Post-traumatic Stress Disorder (PTSD).

	Frequency Model			Dominant Model			Recessive Model		
	b_j_	95%CI	p-value	b_j_	95%CI	p-value	b_j_	95%CI	p-value
Year of publication									
Biallelic approach	−0.0521	−0.1764, 0.0723	0.41	−0.0728	−0.3205, 0.1749	0.56	−0.0132	−0.2193, 0.1928	0.90
Triallelic approach	0.2077	−0.0769, 0.4924	0.15	0.0550	−0.3532, 0.4631	0.79	0.4648	−0.0054, 0.9351	0.50
Males (%)									
Biallelic approach	0.0094	−0.0227, 0.4140	0.57	0.0042	−0.0440, 0.0524	0.86	0.0346	−0.0175, 0.0868	0.19
Triallelic approach	0.0007	−0.0431, 0.0445	0.97	−0.0124	−0.0639, 0.0390	0.64	0.0167	−0.0696, 0.1030	0.70
Mean Age									
Biallelic approach	−0.0110	−0.0431, 0.0212	0.50	0.0046	−0.0561, 0.0652	0.88	−0.0315	−0.1027, 0.0397	0.39
Triallelic approach	−0.0177	−0.0516, 0.0162	0.31	**−0.0324**	**−0.0582, −0.0065**	**0.01**	−0.0259	−0.0964, 0.0446	0.47
European or Caucasian (%)									
Biallelic approach	−0.0048	−0.0126, 0.0030	0.23	−0.0072	−0.0188, 0.0044	0.23	−0.0045	−0.0203, 0.0113	0.57
Triallelic approach	−0.0039	−0.0126, 0.0048	0.38	**−0.0102**	**−0.0178, −0.0025**	**<0.01**	−0.0018	−0.0199, 0.0163	0.85
Afro-Americans and Africans (%)									
Biallelic approach	0.0015	−0.0066, 0.0096	0.72	0.0053	−0.0060, 0.0166	0.36	0.0002	−0.0158, 0.0162	0.98
Triallelic approach	0.0035	−0.0052, 0.0123	0.43	**0.0110**	**0.0033, 0.0187**	**0.005**	−0.0004	−0.0180, 0.0172	0.96
Total Quality Score (TQS)									
Biallelic approach	0.0120	−0.0483, 0.0723	0.70	0.0175	−0.0666, 0.1017	0.69	0.0418	−0.0605, 0.1440	0.42
Triallelic approach	0.1484	−0.0670, 0.3638	0.18	0.0113	−0.2636, 0.2863	0.94	**0.3518**	**0.4017, 0.6619**	**0.03**

**b_j_**: regression coefficient for the moderator variable; **95% CI**: 95% Confident Interval.

### Gene-Environmental (GxE) Interactions

Only six included studies analyzed GxE interactions [16], [17], [19], [20], [50], [51] (Table 1). The environments studied were those of high crime and high unemployment rates [16], adult traumatic events [20], childhood adversities [20], [51] and number of traumatic events experienced [17], [19], [50]. Four studies detected a significant GxE interaction, three of them with the *S* or *S*' allele interacting with the environment [16], [20], [51] and the fourth described an additive GxE interaction with the *L*' (*LA*) allele [19]. Interestingly, the GxE interaction effect, with childhood adversity as the modifying variable, was only described in European Americans but not in Afro-Americans [20], [51]. Two studies did not find a significant GxE interaction for *5-HTTLPR* genotype [17], [50]. The low number of studies of each different environment did not permit a meta-analysis.

## Discussion

To our knowledge, this is the first published meta-analysis of the relationship between *5-HTTLPR* polymorphisms and PTSD. We found no significant relationship between biallelic or triallelic polymorphism and PTSD, using the three possible approaches (allele frequency, dominant and recessive model). Contrary to expectation, the triallelic approach (*S*' and *L*') did not appear to alter the results of the meta-analysis obtained by the biallelic approach (*S* and *L*). There was no apparent publication bias but there was great variability in TQS and in some individual quality characteristics of the included studies. For example, there was a significant main effect when the analysis was restricted to those studies measuring current PTSD (BRM, TFM and TRM) and a significant effect in cohort studies was apparent in TFM and TRM, the study design which is less prone to bias than case-control and cross-sectional ones. Finally, several potential moderators were detected in the meta-regression analyses (mean age of participants, ethnic distribution of the population studied and the TQS) and these significantly modified the results. These results suggest that when more homogeneous groups within studies are considered, thus fulfilling these criteria, potential evidence that PTSD is associated with 5-HTTLPR may indeed emerge and this warrants further research.

There has recently been increasing interest in developing guiding principles for reporting results of different study designs in order to improve the quality of research reports. The STrengthening the REporting of Genetic Association studies (STREGA) Statement was published in 2009 [26] as an extension of the Strengthening the Reporting of Observational Studies in Epidemiology (STROBE) [54] and was specifically designed to enhance the transparency of the reports of genetic association studies. Interestingly, none of the seven studies published since then [17], [45], [46], [48]–[51] has followed these recommendations.

One possible consequence of our results might be that PTSD is not directly associated with 5-HTTLPR. However, several other reasons need to be considered to explain the absence of a significant effect of the *5-HTTLPR* on PTSD, given the increasing evidence suggesting the implication of the 5-HTTLPR polymorphisms and different psychopathological conditions related to stress sensitivity [5]. Firstly, published work in this research area has only considered a third functional allele in the *5-HTTLPR* polymorphisms. Our findings do not support significant differences between the biallelic and the triallelic analyses. Nevertheless, at least ten allelic variants have been described in *5-HTTLPR* polymorphisms in humans [13] and it is not clear whether the magnitude of any association may be affected or moderated by this variability.

Secondly, the lack of a significant association may be due to limited statistical power. Emerging evidence from large genetic studies of other mental disorders suggest the individual effect size of specific alleles may be very small with disorders which are highly polygenic [55]. Consistent with this, findings of other meta-analyses of the same genetic factor with different phenotypes have found a small association. For example, the pooled OR for the *S* allele and unipolar depression is 1.08 (95% CI  = 1.03, 1.12) [56], 1.12 (95% CI  = 1.03, 1.21) for bipolar disorder [57] and 1.18 (95% CI  = 1.02, 1.33) for alcohol dependence [58]. In our meta-analysis, twelve studies were included in the biallelic model with 1,874 PTSD cases but only seven were included in the triallelic with 627 PTSD cases. Therefore, if we consider the different study designs and types of PTSD assessed, it is reasonable to consider the possibility of a lack a statistical power as necessary to detect a true relationship between *5-HTTLPR* and PTSD. If this is the case, new association studies with larger samples are necessary and further meta-analyses should be performed in future to detect a more accurate estimation of a true effect. Another example is related to population stratification. Interestingly, the first published study [18] was performed in an Asian population and has not been replicated and all studies published since have been performed in Caucasian or African or Afro-American populations. As differences in ethnic distribution have been described [8], [11], [13], [16], population stratification should be controlled or stratified in future studies.

Thirdly, *5-HTTLPR* might not have a primary direct effect on PTSD. As PTSD diagnosis requires exposure to an environmental stressor, it is an ideal condition for the investigation of GxE interactions, where the effects of environmental exposure are moderated by genotype [4], [59]. However, only six studies have formally tested GxE interactions in this field [16], [17], [19], [20], [50], [51]. So far, the low number of studies and the high variability of stressors analyzed do not allow formal meta-analyses of these interactions. On the other hand, there may be other interactions underlying the potential role of specific genes in the etiology of PTSD. For example, a gene-gene interaction might also explain the absence of a main genetic effect in PTSD as has been described in neuroticism, where *BDNF Val66Met* interacts with *5-HTTLPR* to influence neuroticism [60]. It is also possible that the relationship between the 5-HTTLPR genotype and PTSD could be mediated by other personality traits as has been suggested in the case of neuroticism as a mediator of the association of the *5-HTTLPR* polymorphism with lifetime major depression [61].

Finally, epigenetic modification offers a promising research area that may clarify the variability of the results obtained in PTSD research as well as in other psychiatric illnesses [62]. There has been an increasing interest in epigenetic factors in psychiatric disorders and this involves the study of inheritable changes in gene expression that occur without changes in the DNA sequence. The level of methylation of *5-HTTLPR* modified the effect of the number of traumatic experiences on the risk of PTSD regardless of the *5-HTTLPR* polymorphisms [50] and it may explain how the environment can modify gene expression regardless of the primary genetic sequence by changing the accessibility of information printed on the DNA [63].

We acknowledge some limitations of this meta-analysis in relation to interpretation of the findings. Some studies could not be included in the final analysis because of incomplete data. However, it is unlikely that their inclusion would have affected our main results due to the small sample size and their lower quality characteristics. Errors or bias in the design of or in the statistical tests in the primary observational studies could potentially affect the results of our meta-analysis. Although 61% of the included studies performed a controlled analysis for potential confounding variables, our meta-analyses were performed using the crude OR as the diversity of confounding variables did not allow the use of the adjusted OR. Although this is a general limitation of meta-analysis in observational studies, careful analyses of the individual quality aspects and the different study designs of the primary studies were performed to explore the moderate/large levels of heterogeneity detected.

Of particular concern is the heterogeneity resulting from the varying ethnic distribution within studies and its potential confounding effect on the pooled effect size. To analyze this particular aspect, different approaches were considered including a subgroup analysis of the assessment of ethnicity and specific meta-regression analyses of the effect of the percentage of different ethnic groups (European/Caucasian or Afro-Americans/Africans). Our results suggest that this ethnic distribution should be considered as an important modifying variable in future research.

On the other hand, several strengths of our meta-analysis deserve recognition. Firstly, although a TQS was calculated and used in the meta-regression analyses, the relevant methodological aspects were assessed individually and the influence on the magnitude of the effect was explored as the TQS (as a summation of points) giving equal weight to all characteristics [28]. Secondly, we analyzed three inheritance models, as it was not yet clear which one better represents the association between the *5-HTTLPR* poylymorphisms and PTSD as well as performing two allelic studies (biallelic and triallelic). Given that there was little variation according to the model or the approach studied, this supports our view that our findings are indeed robust. Finally, we have followed current guidelines for design and reporting of systematic reviews of genetic association studies [24], [27], [34]. Nevertheless, these guidelines should be adapted to the specific characteristics of psychiatric genetics as has recently been suggested for the adaptation of the Meta-analysis of Observational Studies in Epidemiology (MOOSE) guidelines [64] to be used in psychiatric epidemiology [65].

### Implications

Although current evidence does not support a direct main effect of the *5-HTTLPR* polymorphisms on PTSD, GxE interactions and epigenetic modulation offer promising research areas in the near future. Further studies of possible genetic associations between *5-HTTLPR* and PTSD are needed to clarify this relationship and the role of the potential moderators detected. Special attention should be paid to some characteristics specifically in relation to PTSD research, including exposure of controls to the same stressor as PTSD cases, type of PTSD assessed, psychiatric comorbidity and population stratification. These new studies, in addition to those already published on the effect of GxE interactions and on new potential environmental factor candidates to interact with the *5-HTTLPR* polymorphisms, may help to clarify the role of this and other genetic factors in PTSD.
